# Intramolecular Chain Hydrosilylation of Alkynylphenylsilanes Using a Silyl Cation as a Chain Carrier

**DOI:** 10.3390/molecules21080999

**Published:** 2016-08-01

**Authors:** Hidekazu Arii, Kenichi Nakabayashi, Kunio Mochida, Takayuki Kawashima

**Affiliations:** 1 Faculty of Education, University of Miyazaki, 1-1 Gakuen Kibanadai Nishi, Miyazaki, 889-2192 Miyazaki, Japan; nakabys@cc.miyazaki-u.ac.jp; 2 Department of Chemistry, Gakushuin University, 1-5-1 Mejiro, Toshima-ku, 171-8588 Tokyo, Japan; kunio.mochida@gakushuin.ac.jp; 3 Graduate School of Science and Technology, Gunma University, 1-5-1 Tenjin-cho, Kiryu, 376-8515 Gunma, Japan

**Keywords:** benzosilole, silyl cation, hydrosilylation, metal free

## Abstract

Diorganyl[2-(trimethylsilylethynyl)phenyl]silanes **1a**–**c** and methyl-substituted phenylsilanes **1d** and **1e** were treated with a small amount of trityl tetrakis(pentafluorophenyl)borate (TPFPB) as an initiator in benzene to afford the corresponding benzosiloles (**2a**–**e**) in moderate to good yields. However, no reaction was observed for the reaction using [2-(1-hexynyl)phenyl]diisopropylsilane **lf**. The methyl substituent was tolerated under the reaction conditions and increased the yield of the corresponding benzosilole depending on the substitution position. From the result using **1f**, the current reaction was found to require the trimethylsilyl group, which can stabilize intermediary alkenyl carbocations by the β-silyl effect. The current reaction can be considered an intramolecular chain hydrosilylation of alkynylarylsilanes involving silyl cations as chain carriers. Therefore, the silyl cations generated by hydride abstraction from hydrosilanes **1** with the trityl cation causes intramolecular electrophilic addition to the C-C triple bond to form ethenyl cations, which abstract a hydride from **1** to afford benzosiloles **2** with the regeneration of the silyl cations.

## 1. Introduction

A benzosilole is an attractive compound due to its emission property and potential use as of optical materials [1,2,3,4]. These features are associated with the low-lying LUMOs of the siloles, which originate from orbital interaction between the σ* orbital of the silylene moiety and the π* orbital of the butadiene moiety [5]. The popular synthetic routes to benzo- and dibenzosiloles involve intra- and intermolecular cyclization reactions with transition metal catalysts [6,7,8,9], and the use of a chiral supporting ligand enables the synthesis of siloles with a chiral silicon center [10,11]. In particular, direct Si–C or C–H activation is a powerful method that does not require an activated functional group on the aromatic ring [12,13,14,15,16,17]. In non-transition metal systems, 2-ethynylphenylsilane derivatives have been cyclized to afford the corresponding benzosiloles using various reactants, such as lithium naphthalenide [18], Lewis acids [19,20] and KH (Equation (1)) [21]. The radical reaction that is initiated by *tert*-butylhydroperoxide (TBHP) resulted in the formation of dibenzosiloles from (2-biphenyl)diphenylsilanes (Equation (2)) [22]. The uses of KH and TBHP produce the corresponding active silicon species (i.e., a pentacoordinated hydridosilicate and a silyl radical, respectively). We have synthesized dibenzosilole by a sila-Friedel-Crafts reaction mediated by a silyl cation (Equation (3)) [23,24].

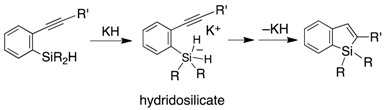
(1)

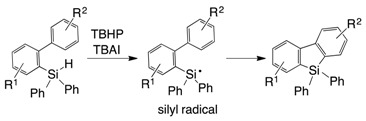
(2)

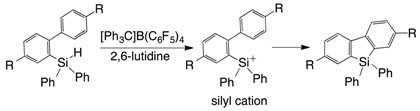
(3)

We have extended silyl cation chemistry to the synthesis of silacyclic compounds and reported the synthesis of 1,2-dihydro-2-silanaphthalenes using alkynes [25,26]. The intramolecular addition of a silyl cation toward a C–C triple bond rather than an intermolecular one is expected to lead to a benzosilole by an appropriate reaction of the resulting ethenyl carbocation. Herein, we report the intramolecular chain hydrosilylation of diorganyl[2-(trimethylsilylethynyl)phenyl]silanes with trityl tetrakis(pentafluorophenyl)borate (TPFPB) as an initiator.

## 2. Results and Discussion

Diorganyl[2-(trimethylsilylethynyl)phenyl]silanes **1a**–**c** were reacted with a small amount (1–4 mol %) of TPFPB in benzene to afford the corresponding benzosiloles **2a**–**c** in low to moderate yields (Table 1, entries 1–3). The intramolecular chain hydrosilylation of **1** was achieved using TPFPB as an initiator. However, this reaction was accompanied with the formation of unidentified oligomers, which appear to be formed by silyl cation-induced alkyne polymerization. The amount of TPFPB and solvent were optimized using **1b**, and therefore, 1 mol % TPFPB in benzene was determined to provide the best yield of 75% (Table 1, entry 5). The conversion of **1b** to silole **2b** required a longer reaction time (30 min in 1 mol % TPFPB (Table 1, entry 5) compared to 15 min in 3 mol % TPFPB (entry 4) and 5 min in 4 mol % TPFPB (entry 2)) with a decrease in the dose of the trityl cation initiator. However, the yield of **2b** increased from 61% to 75% due to the preference of the desired hydrosilylation over the competing oligomerization. The syntheses of benzosiloles from 2-alkynylphenylsilane derivatives in transition metal-free systems have been achieved using AlCl_3_ and KH to activate the Si-H and/or C–C triple bonds and to generate the pentacoordinated hydridosilicate, respectively [19,21]. In this system, the silyl cation plays an important role in the reaction and promotes the hydrosilylation by electrophilic addition to the C–C triple bond.

The scope and limitations of the intramolecular chain hydrosilylation of **1** are summarized as follows. Under the optimized conditions using 1 mol % of TPFPB, the isolated yields of **2b** and **2c** were increased (entries 5 and 10) even though the yield of **2a** bearing the sterically smaller methyl groups on the silicon center was barely affected by the dose of initiator (entry 9). The reactions using methyl-substituted silanes **1d** and **1e** also afforded the corresponding benzosiloles **2d** and **2e** in 72% and 81% yields, respectively (entries 11 and 12). It is most likely that the relatively good yield of **2e** may be caused by stabilization due to hyperconjugation of the 5-methyl group with the intermediary ethenyl carbocation. The reaction using silane **1f** bearing a 1-hexynyl group rather than a trimethylsilylethynyl group did not afford the corresponding silole **2f**, and nearly all of **1f** was recovered (entry 13). This result may be due to the intermediary ethenyl carbocation that was derived from **1f** being less stable than those derived from **1a**–**e**, which are stabilized by double β-silyl effects. Therefore, the trimethylsilyl group on the alkynyl group was essential for the current reaction.

**Scheme 1:**
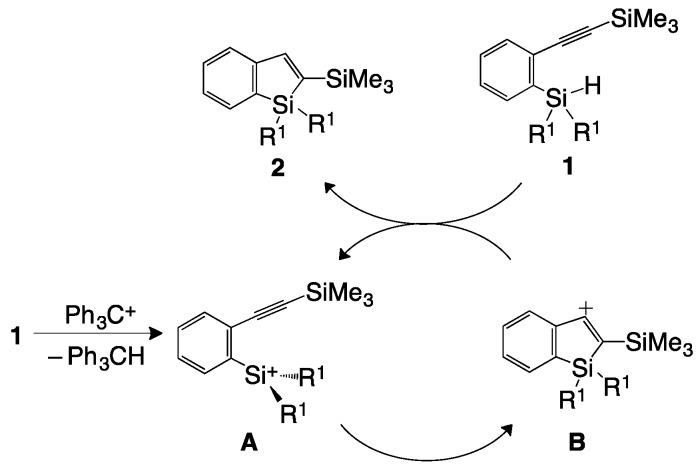
Plausible mechanism of intramolecular chain hydrosilylation.

The reaction mechanism of the intramolecular chain hydrosilylation is described in Scheme 1. First, the trityl cation acts as an initiator to abstract the hydride from the Si–H bond of **1**, resulting in the generation of silyl cation **A**. Next, the intramolecular electrophilic addition of the silyl cation moiety of **A** to the C–C triple bond produces ethenyl carbocation **B**, which is stabilized by the β-silyl effect of the trimethylsilyl group. Finally, the hydride abstraction of **B** from another **1** affords benzosilole **2** and regenerates intermediate **A** [27,28,29], which acts as a chain carrier.

## 3. Experimental Section

General Procedure: All experiments were carried out using standard vacuum line and Schlenk techniques in an Ar atmosphere or dry box. All the reagents were of the highest grade available and were used without further purification. All solvents used for the syntheses were distilled according to the general procedure. [Ph_3_C]B(C_6_F_5_)_4_ [30], [2-(2-bromophenyl)ethynyl]trimethylsilane derivatives [31], 2-(1-hexynyl)bromobenzene [21], **1a** [19] and **1c** [21] were synthesized according to the previously reported methods. The NMR spectral measurements were performed on an Agilent 400-MR NMR (Agilent Technologies Co., Santa Clara, CA, USA) or a Bruker AV400M spectrometers (Bruker Co., Billerica, MA, USA). The ^1^H and ^13^C chemical shifts are reported relative to the residual protonated solvent and the solvent, respectively, according to the literature [32]. High-resolution mass spectrometry was measured by a JEOL GCMATE II (JEOL Ltd., Tokyo, Japan) or JMS-700N (JEOL Ltd.) operating by electron impact ionization (EI). Gel permeation liquid chromatography (GPLC) was performed by a Japan Analytical Industry LC-918 (Japan Analytical Industry Co., Ltd., Tokyo, Japan) using chloroform as an eluent.

### Preparation of Compounds

Silanes **1**. To a corresponding bromo compound (1.3 mmol) in hexane 8 mL were added 1.6 M pentane solution of *tert*-BuLi (0.84 mL, 1.4 mmol) and *N,N,N’,N’*-tetramethylethylenediamine (0.23 g, 2.0 mmol) at −80 °C, and the solution was stirred for 20 min keeping the temperature below −70 °C. To the solution was added *i*-Pr_2_SiHCl (0.20 g, 1.4 mmol) at −70 °C, the solution was stirred and slowly warmed to room temperature. The reaction mixture was quenched with 5% NH_4_Cl aqueous solution. The mixture was extracted with hexane 20 mL two times, and the organic layer was dried over anhydrous sodium sulfate. The filtrate was concentrated under reduced pressure to remove volatiles, and the residue was purified by silica gel column (eluent: hexane). Further purification was carried out by GPLC to obtain **1** as a colorless liquid. 

*Diisopropyl[(2-trimethylsilylethynyl)phenyl]silane* (**1b**): 75%. ^1^H-NMR (CDCl_3_, 400 MHz): δ 7.50–7.46 (m, 2H, ArH), 7.32–7.24 (m, 2H, ArH), 4.01 (t, *J* = 4.0 Hz, 1H, SiH), 1.49–1.39 (m, 2H, *i*-Pr), 1.10 (d, *J* = 7.2 Hz, 6H, *i*-Pr), 0.98 (d, *J* = 7.6 Hz, 6H, *i*-Pr), 0.24 (s, 9H, SiMe_2_). ^13^C-NMR (CDCl_3_, 100 MHz): δ 138.1, 136.5, 132.6, 128.9, 128.8, 127.4, 106.6, 96.1, 19.12, 19.07, 11.1, −0.22. HRMS (EI) *m/z*: [M]^+^ Calcd for C_17_H_28_Si, 288.1730; Found, 288.1729.

*Diisopropyl[4-methyl-2-(trimethylsilylethynyl)phenyl]silane* (**1d**): Yield 69%. ^1^H-NMR (CDCl_3_, 400 MHz): δ 7.39 (d, *J* = 7.6 Hz, 1H, ArH), 7.30 (d, *J* = 1.2 Hz, 1H, ArH), 7.11 (dd, *J* = 8.0 Hz, *J* = 1.2 Hz, 1H, ArH), 3.98 (t, *J* = 4.0 Hz, 1H, SiH), 2.34 (s, 3H, ArMe), 1.50–1.40 (m, 2H, *i*-Pr), 1.11 (d, *J* = 7.6 Hz, 6H, *i*-Pr), 1.10 (d, *J* = 7.2 Hz, 6H, *i*-Pr), 0.24 (s, 9H, SiMe_3_). ^13^C-NMR (CDCl_3_, 100 MHz): δ 138.0, 137.4, 137.2, 132.6, 129.6, 125.8, 106.9, 95.1, 21.5, 19.20, 19.13, 11.2, −0.18. HRMS (EI) *m/z*: [M]^+^ Calcd for C_18_H_30_Si_2_, 302.1886; Found, 302.1909.

*Diisopropyl[5-methyl-2-(trimethylsilylethynyl)phenyl]silane* (**1e**): 68%. ^1^H-NMR (CDCl_3_, 400 MHz): δ 7.46 (dd, *J* = 7.2 Hz, *J* = 1.2 Hz, 1H, ArH), 7.41 (d, *J* = 7.2 Hz, 1H, ArH), 7.28 (td, *J* = 7.6 Hz, *J* = 1.6 Hz, 1H, ArH), 7.22 (td, *J* = 7.6 Hz, *J* = 1.6 Hz, 1H, ArH), 4.02 (t, *J* = 4.0 Hz, 1H, SiH), 2.41 (t, *J* = 7.2 Hz, 2H, *n*-Bu), 1.62–1.32 (m, 6H, *i*-Pr and *n*-Bu), 1.10 (d, *J* = 7.6 Hz, 6H, *i*-Pr), 0.98 (d, *J* = 7.2 Hz, 6H, *i*-Pr), 0.95 (t, *J* = 7.2 Hz, 3H, *n*-Bu). ^13^C-NMR (CDCl_3_, 100 MHz): δ 137.4, 136.4, 132.1, 130.0, 128.8, 126.4, 92.4, 82.1, 30.7, 22.1, 19.24, 19.12, 13.6, 11.1. HRMS (EI) *m/z*: [M]^+^ Calcd for C_18_H_28_Si, 272.1960; Found, 272.1958.

*[2-(1-Hexynyll)phenyl]diisopropylsilane* (**1f**): Yield 55%. ^1^H-NMR (CDCl_3_, 400 MHz): δ 7.38 (d, *J* = 7.6 Hz, 1H, ArH), 7.34 (s, 1H, ArH), 7.10 (d, *J* = 7.6 Hz, 1H, ArH), 4.00 (t, *J* = 4.0 Hz, 1H, SiH), 2.31 (s, 3H, ArMe), 1.47–1.36 (m, 2H, *i*-Pr), 1.10 (d, *J* = 7.2 Hz, 6H, *i*-Pr), 0.98 (d, *J* = 7.2 Hz, 6H, *i*-Pr), 0.24 (s, 9H, SiMe_3_). ^13^C-NMR (CDCl_3_, 100 MHz): δ 138.7, 136.7, 134.4, 133.3, 128.8, 128.5, 106.8, 95.6, 21.1, 19.12, 19.09, 11.1, −0.21. HRMS (EI) *m/z*: [M]^+^ Calcd for C_18_H_30_Si_2_, 302.1886; Found, 302.1883.

Benzosiloles **2.** To trityl tetrakis(pentafluorophenyl)borate (TPFPB, 1.0 mg, 1.0 μmol) in benzene (0.5 mL) was added a benzene solution (1.5 mL) of hydrosilanes **1** (0.10 mmol) at room temperature under Ar atmosphere, and the resulting solution was stirred at room temperature. After the reaction mixture was quenched with 2,6-lutidine (2 μL) and H_2_O, and then the organic layer was extracted. After extraction with hexane two times, the organic layers were combined and dried over anhydrous sodium sulfate, and then the filtrate was evaporated under reduced pressure to remove volatiles. Purification was carried out by GPLC to remove polymeric materials.

*1,1-Dimethyl-2-trimethylsilyl-1-silaindene* (**2a**): 34%. ^1^H-NMR data are consisted with those reported previously [19].

*1,1-Diisopropyl-2-trimethylsilyl-1-silaindene* (**2b**): 75%. ^1^H-NMR (CDCl_3_, 400 MHz): δ 7.61 (s, 1H, ArCH=C), 7.51 (d, *J* = 6.8 Hz, 2H, ArH), 7.32 (td, *J* = 7.6 Hz, *J* = 1.2 Hz, 1H, ArH), 7.27 (d, *J* = 6.4 Hz, 1H, ArH), 7.19 (td, *J* = 7.6 Hz, *J* = 1.2 Hz, 1H, ArH), 1.35–1.25 (m, 2H, *i*-Pr), 1.07 (d, *J* = 7.6 Hz, 6H, *i*-Pr), 0.93 (d, *J* = 7.6 Hz, 6H, *i*-Pr), 0.19 (s, 9H, SiMe_2_). ^13^C-NMR (CDCl_3_, 100 MHz): δ 158.2, 151.1, 143.1, 137.4, 132.6, 129.4, 126.7, 123.9, 19.98, 19.96, 11.2, −0.16. HRMS (EI) *m/z*: [M]^+^ Calcd for C_17_H_28_Si, 288.1730; Found, 288.1736.

*1,1-Diphenyl-2-trimethylsilyl-1-silaindene* (**2c**): 55%. ^1^H-NMR data are consisted with those reported previously [21].

*1,1-Diisopropyl-5-methyl-2-trimethylsilyl-1-silaindene* (**2d**): Yield 72%. ^1^H-NMR (CDCl_3_, 400 MHz): δ 7.57 (s, 1H, ArCH=C), 7.40 (d, *J* = 6.8 Hz, 2H, ArH), 7.11 (s, 1H, ArH), 7.02 (d, *J* = 6.8 Hz, 1H, ArH), 2.35 (s, 3H, ArMe), 1.34–1.22 (m, 2H, *i*-Pr), 1.06 (d, *J* = 7.2 Hz, 6H, *i*-Pr), 0.93 (d, *J* = 7.2 Hz, 6H, *i*-Pr), 0.19 (s, 9H, SiMe_2_). ^13^C-NMR (CDCl_3_, 100 MHz): δ 158.2, 151.6, 143.3, 139.3, 133.6, 132.5, 127.5, 125.0, 21.5, 18.01, 17.99, 11.2, −0.16. HRMS (EI) *m/z*: [M]^+^ Calcd for C_18_H_30_Si_2_, 302.1886; Found, 302.1911.

*1,1-Diisopropyl-6-methyl-2-trimethylsilyl-1-silaindene* (2e): Yield 81%. ^1^H-NMR (CDCl_3_, 400 MHz): δ 7.58 (s, 1H, ArH), 7.32 (s, 1H, ArH), 7.18–7.09 (m, 2H, ArH), 2.36 (s, 3H, ArMe), 1.36–1.22 (m, 2H, *i*-Pr), 1.07 (d, *J* = 7.2 Hz, 6H, *i*-Pr), 0.94 (d, *J* = 7.6 Hz, 6H, *i*-Pr), 0.19 (s, 9H, SiMe_3_). ^13^C-NMR (CDCl_3_, 100 MHz): δ 158.0, 148.7, 141.3, 137.6, 136.2, 133.6, 130.0, 123.6, 21.4, 17.98, 17.9, 11.2, –0.13. HRMS (EI) *m/z*: [M]^+^ Calcd for C_18_H_30_Si_2_, 302.1886; Found, 302.1863.

The ^1^H- and ^13^C-NMR spectral charts of all new compounds **1** and **2** are summarized in supplementary materials.

## 4. Conclusions

In conclusions, we achieved the intramolecular chain hydrosilylation of **1** to synthesize benzosiloles **2**. The hydrosilylation proceeded under mild conditions with a small amount of TPFPB as an initiator and no additives. In this reaction, the silyl cation plays an important role as a chain carrier, which is different from the reactions involving the hydridosilicate and silyl radical.

## Figures and Tables

**Table 1:**
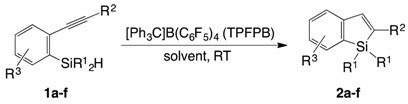
Intramolecular chain hydrosilylation of **1a**–**f** to **2a**–**f**.

Entry		R^1^	R^2^	R^3^	Solvent	TPFPB/mol %	Time/min	Yield of 2/% ^a^
1	**1a**:	Me	TMS	H	benzene	4	5	37 (**2a**)
2	**1b**:	*i*-Pr	TMS	H	benzene	4	5	61 (**2b**)
3	**1c**:	Ph	TMS	H	benzene	4	5	21 (**2c**)
4	**1b**:	*i*-Pr	TMS	H	benzene	3	15	70 (**2b**)
5	**1b**:	*i*-Pr	TMS	H	benzene	1	30	75 (**2b**)
6	**1b**:	*i*-Pr	TMS	H	toluene	1	30	72 (**2b**)
7	**1b**:	*i*-Pr	TMS	H	mesitylene	1	30	70 (**2b**)
8	**1b**:	*i*-Pr	TMS	H	CH_2_Cl_2_	1	30	60 (**2b**)
9	**1a**:	Me	TMS	H	benzene	1	30	34 (**2a**)
10	**1c**:	Ph	TMS	H	benzene	1	30	55 (**2c**)
11	**1d**:	*i*-Pr	TMS	4-Me	benzene	1	30	72 (**2d**)
12	**1e**:	*i*-Pr	TMS	5-Me	benzene	1	50	81 (**2e**)
13	**1f**:	*i*-Pr	*n*-Bu	H	benzene	1	720	0 (**2f**) ^b^

^a^ Isolated yields based on **1**; ^b^ Nearly all of **1f** was recovered.

## Supplementary Materials

The ^1^H- and ^13^C-NMR spectral charts of **1b**, **1d**, **1e**, **1f**, **2b**, **2d**, and **2e** can be accessed at http://www.mdpi.com/1420-3049/21/8/999/s1.
