# Supplementary material for: Intramolecular Chain Hydrosilylation of Alkynylphenylsilanes Using a Silyl Cation as a Chain Carrier
Source: Molecules. 2016 Aug 1;21(8):999. doi: 10.3390/molecules21080999 (PMC6273890; doi:10.3390/molecules21080999)
Supplement: Supplementary file 1 [file molecules-21-00999-s001.pdf]

Molecules **2016**, *21*, 999; doi:10.3390/molecules21080999

Supplementary Materials: Intramolecular Chain Hydrosilylation of Alkynylphenylsilanes Using a Silyl Cation as a Chain Carrier

Hidekazu Arii, Kenichi Nakabayashi, Kunio Mochida and Takayuki Kawashima

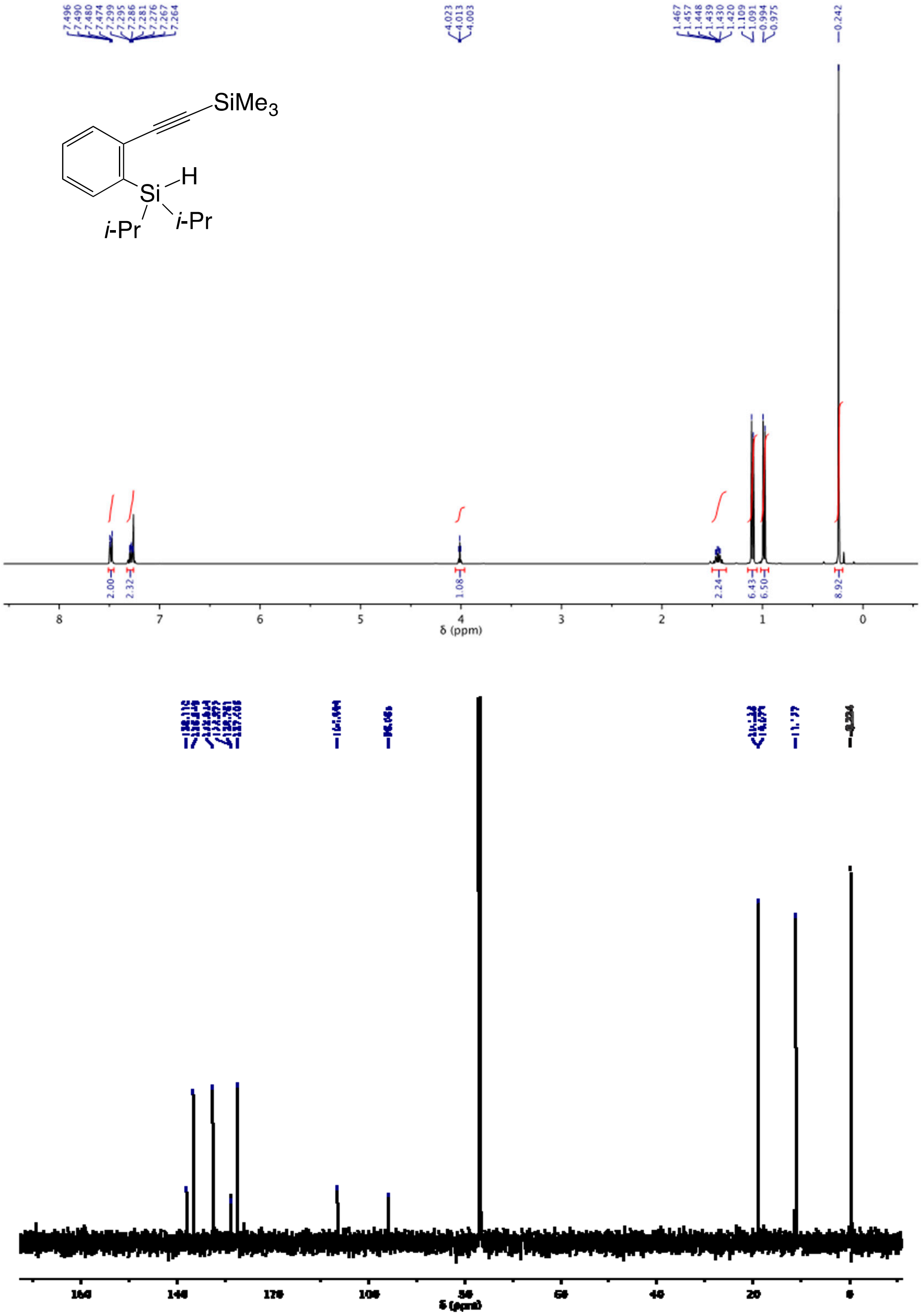

Figure S1. ^{1}H- and ^{13}C-NMR spectra of **1b**.

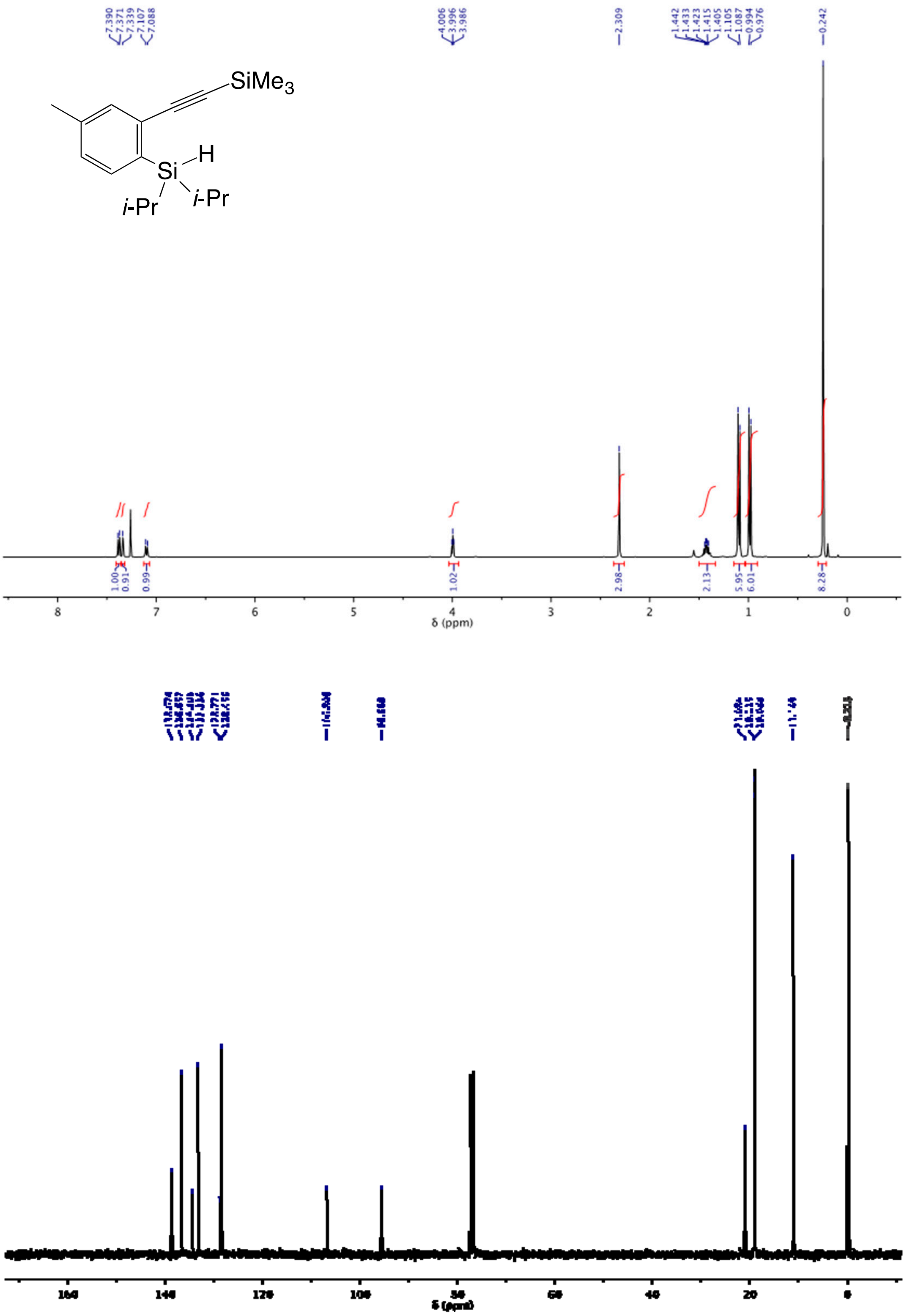

Figure S2. ^{1}H- and ^{13}C-NMR spectra of **1d**.

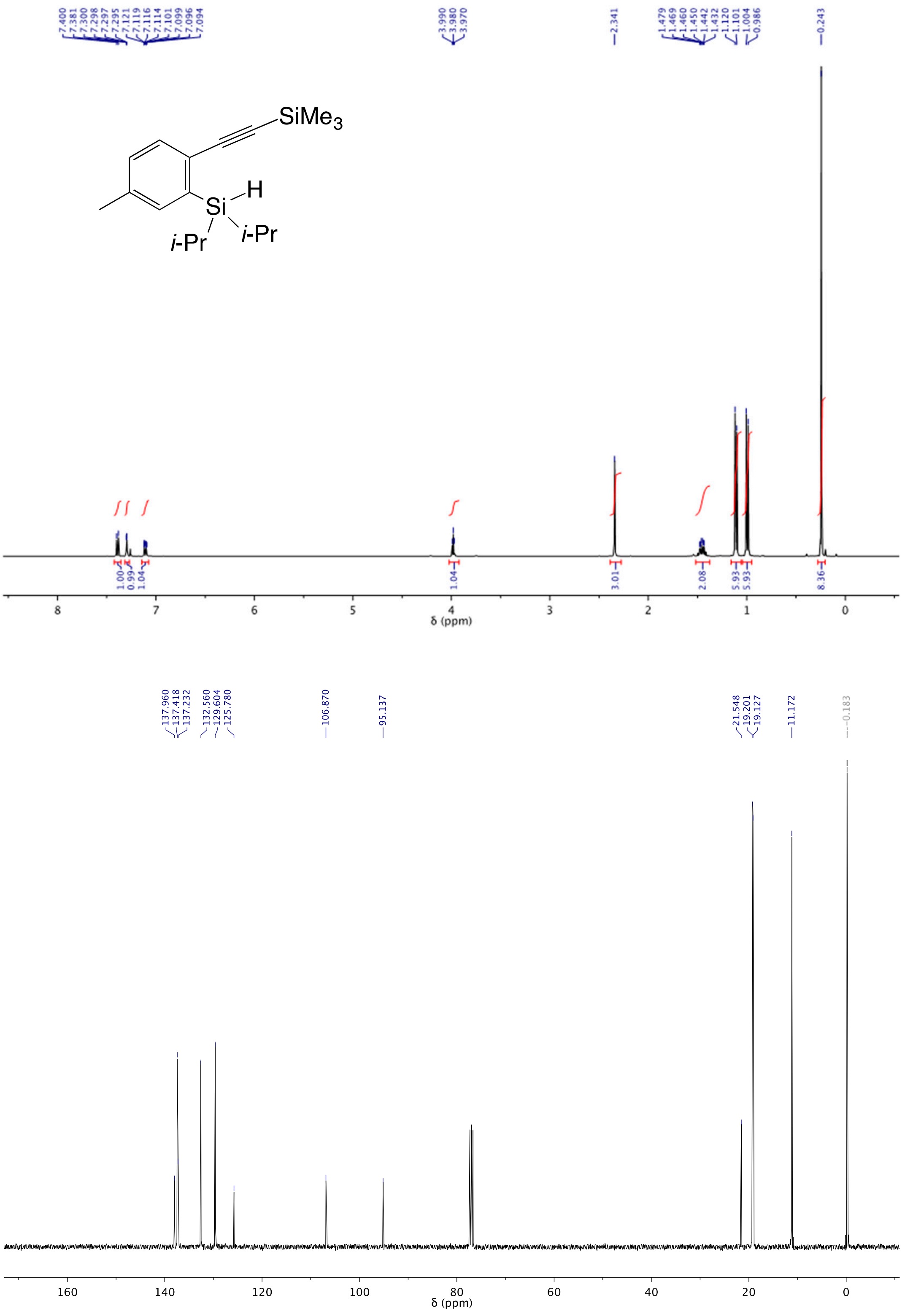

Figure S3. ¹H- and ¹³C-NMR spectra of **1e**.

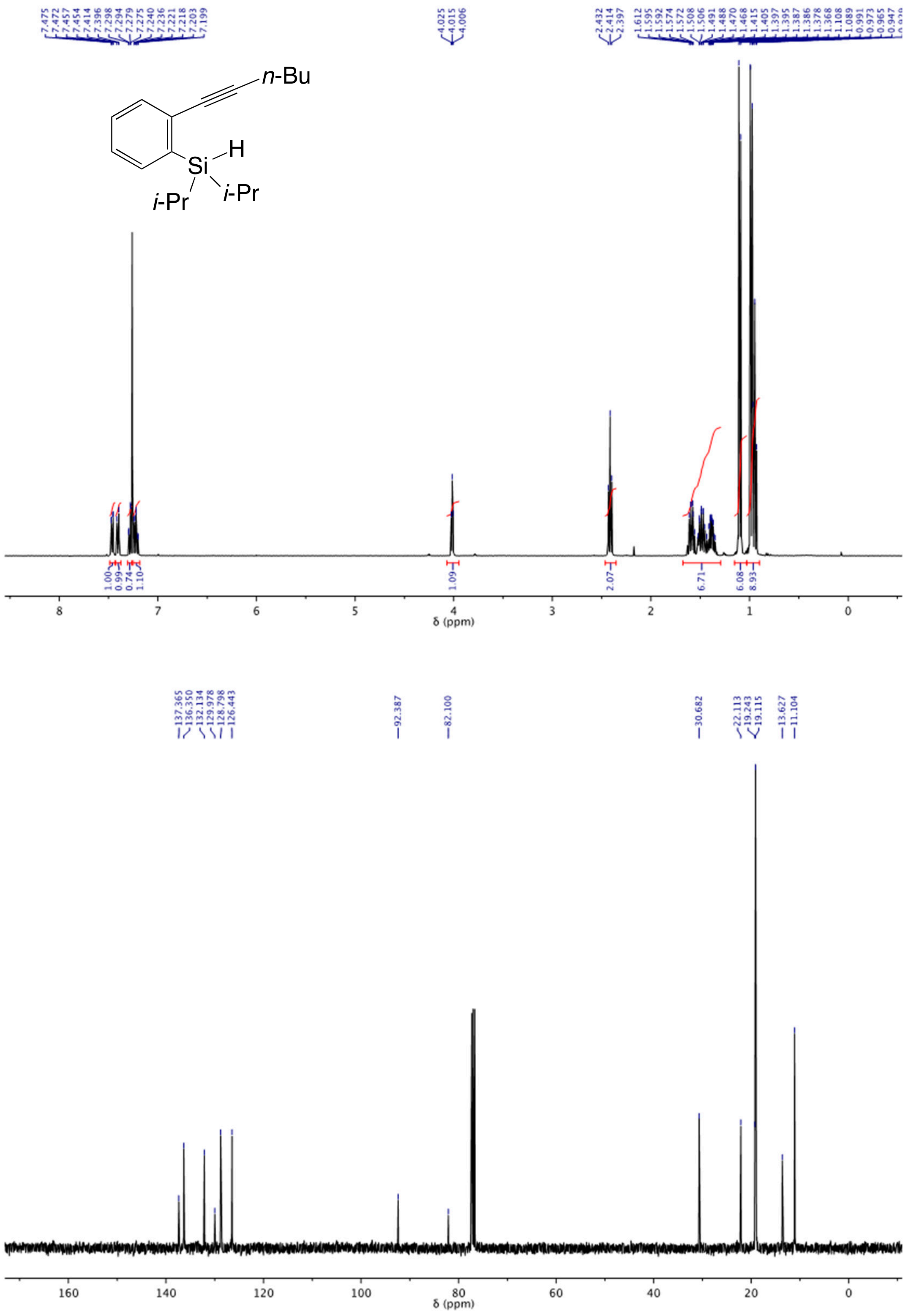

Figure S4. ^{1}H- and ^{13}C-NMR spectra of **1f**.

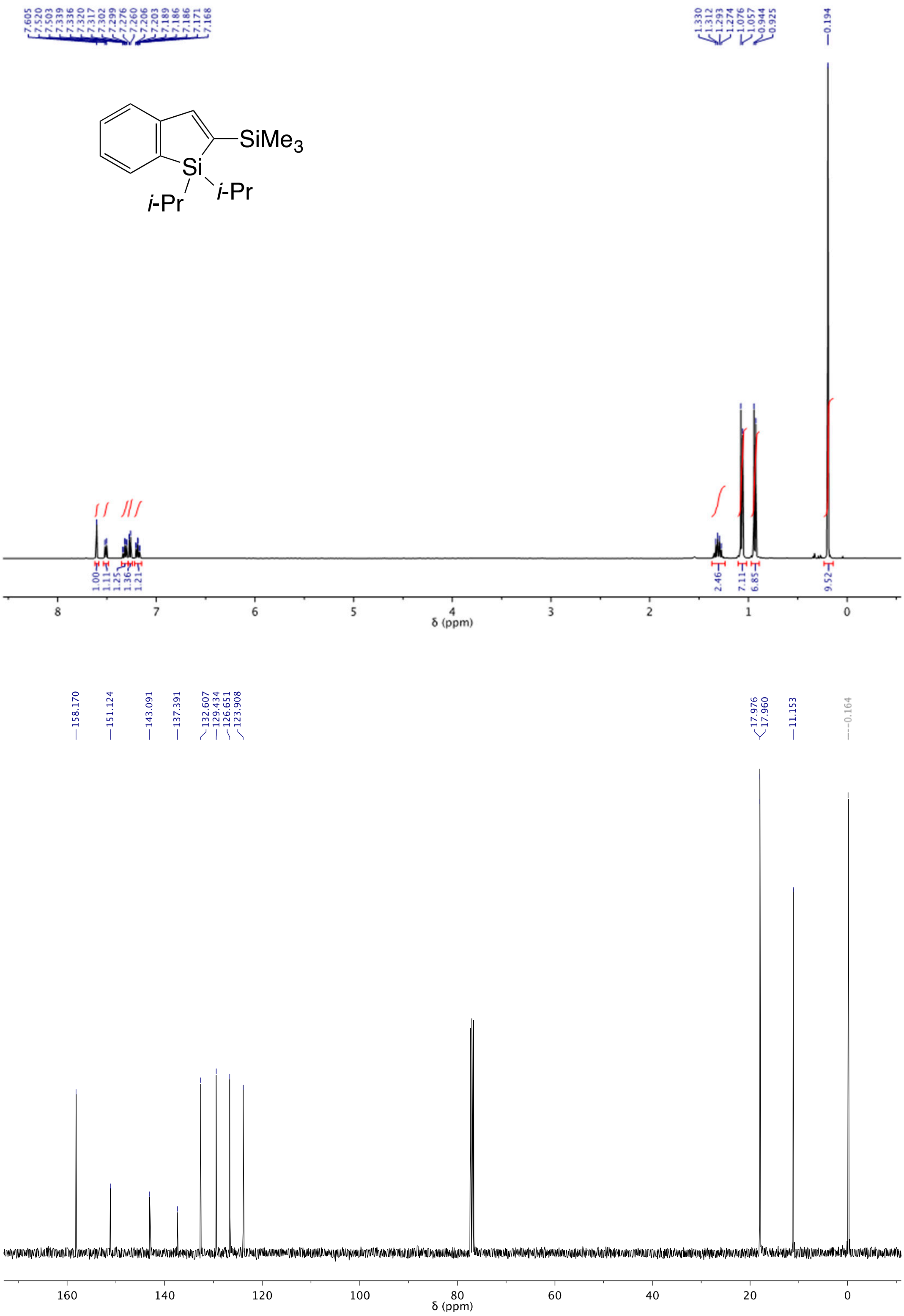

Figure S5. ^{1}H- and ^{13}C-NMR spectra of **2b**.

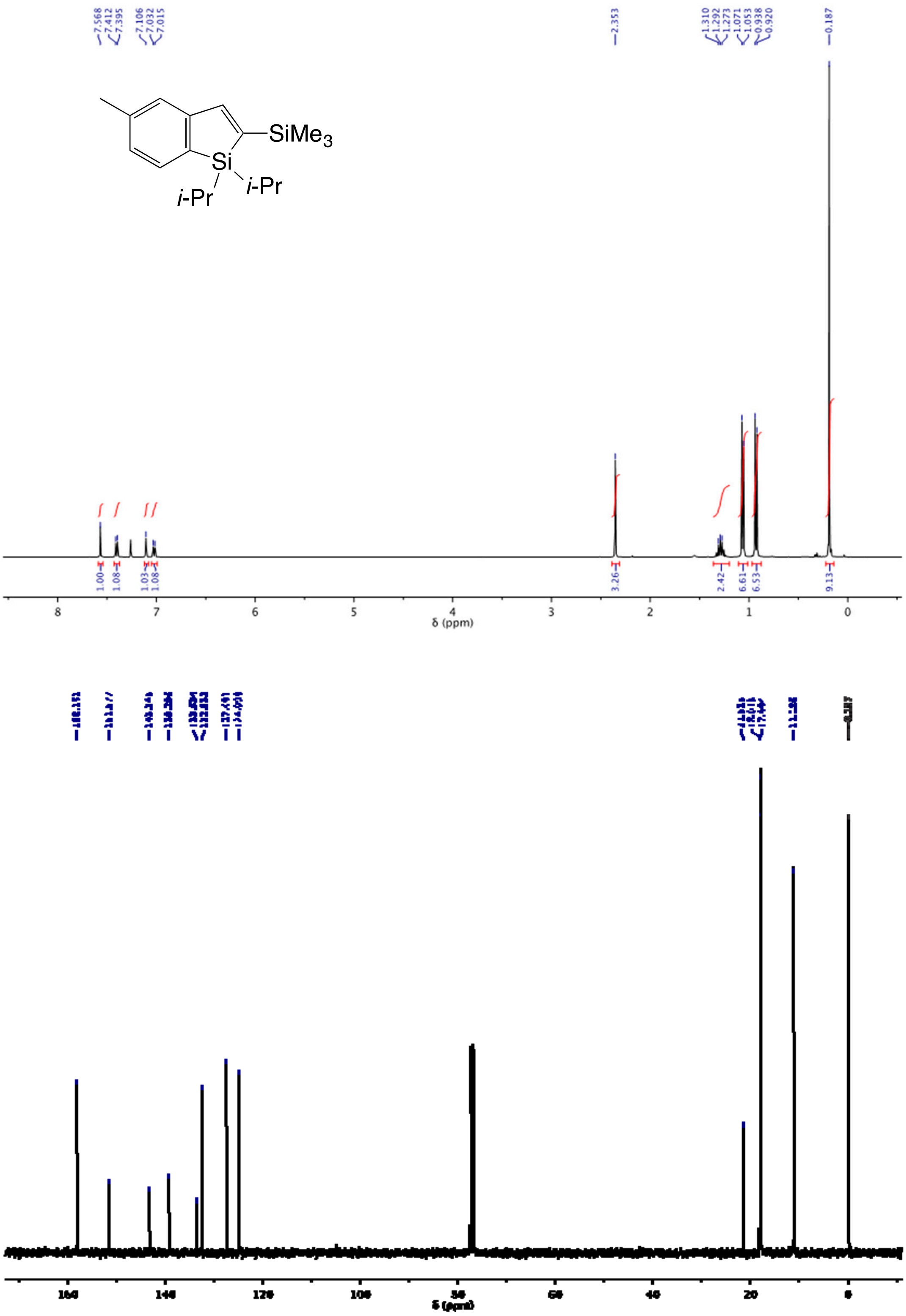

Figure S6. ^{1}H- and ^{13}C-NMR spectra of **2d**.

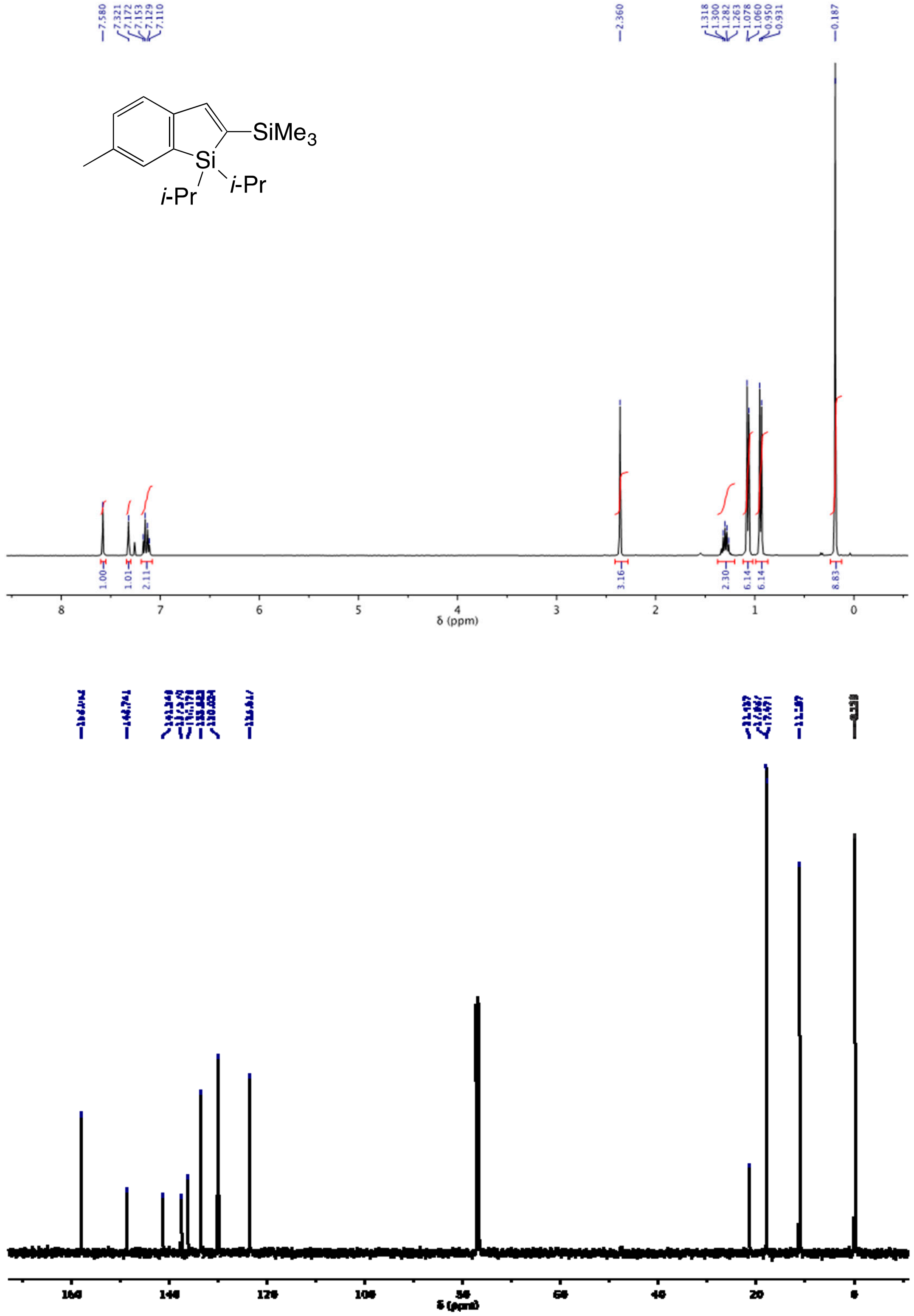

Figure S7. ^{1}H- and ^{13}C-NMR spectra of **2e**.
